# Case report: Mullerian cyst of mediastinum: report of two cases

**DOI:** 10.3389/fonc.2024.1431454

**Published:** 2024-11-13

**Authors:** Aliye Abulizi, Yuan Yang, Peng An, Ping Gao

**Affiliations:** ^1^ Department of Radiology, Postgraduate Union training base of Xiangyang No.1 People's Hospital, School of Medicine, Wuhan University of Science and Technology, Xiangyang, China; ^2^ Department of Radiology, Hubei Clinical Research Center of Parkinson’s Disease, Xiangyang Key Laboratory of Movement Disorders, Xiangyang No.1 People’s Hospital, Hubei University of Medicine, Xiangyang, Hubei, China

**Keywords:** Mullerian cyst, Mediastinal Cyst, tomography, pathological diagnosis, differential diagnosis

## Abstract

Posterior mediastinal Mullerian cyst is rare. Due to its special location, it is easy to be misdiagnosed clinically, imaging and pathologically. Imaging is often misdiagnosed as a bronchial cyst or neurogenic tumor. The postoperative misdiagnosis rate is high and it is easy to be misdiagnosed as a bronchogenic cyst. Careful histopathological observation and necessary immunohistochemical marker staining of the resected specimens, such as PAX 8, WT1, ER and PR positivity, can confirm the diagnosis and avoid misdiagnosis of other lesions. This article introduces the clinical, imaging characteristics, histopathological morphology, and immunohistochemical characteristics of 2 cases of posterior mediastinal Mullerian duct cysts. To explore the clinicopathological characteristics, diagnosis and differential diagnosis of posterior mediastinal Mullerian duct cysts.

## Introduction

Common mediastinal cysts mainly include bronchial cysts, pericardial cysts, thymic cysts, etc. Posterior mediastinal Mullerian cyst is a unique mediastinal cyst first reported by Hattori in 2005 (1), with the histological morphology and immunophenotype of Mullerian differentiation.The reported cases of mediastinal Mullerian cysts so far are all female patients, and the most common age range is 40 to 60 years old (2). Half of the patients are asymptomatic, and some patients have cough or chest pain as the main symptoms.The main treatment for posterior mediastinal Mu llerian cysts is surgical resection. The patient’s postoperative prognosis is good, and no recurrence cases have been reported. However, the long-term prognosis is unclear and requires long-term follow-up observation.

## Case report

**Figure 1:**
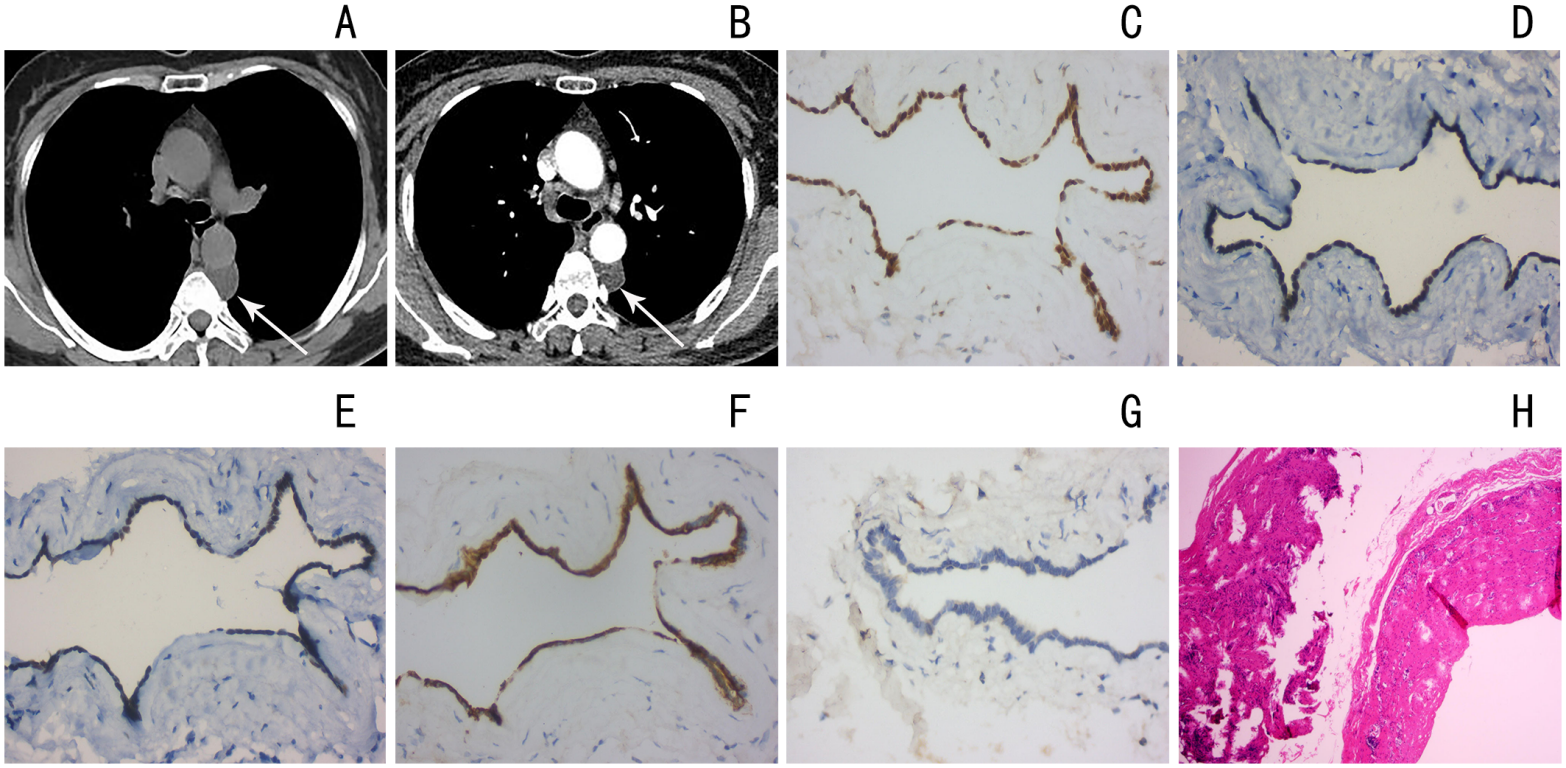
**(A)** Chest CT shows nodular low density in the left posterior mediastinum with clear boundary (arrow) **(B)** chest CT shows nodular low density in the left posterior mediastinum, and mild enhancement on contrast-enhanced scan (arrow) **(C–G)** Estrogen, paired box gene 8(PAX8),Wilms’ tumor protein 1(WT1), protein kinase c (PKC) positive, ki-67 low proliferation **(H)** Pathologic examination of tissue confirmed typical MCS histological features (hematoxylin and eosin stain).

Case 1, female, 47 years old, had intermittent low back pain for more than three months. She was admitted to the hospital on May 21, 2020 because of”tension headache after surgery for right breast fibroma”and had no other clinical symptoms. The patient has a history of hypertension for many years and has a body mass index of 21.8kg/m2. Chest CT showed a low-density mass at the left anterior edge of the T4-5 vertebral body in the posterior mediastinum with a clear boundary and a CT value of about 20Hu (**Figure 1A**); the contrast-enhanced scan showed no obvious enhancement (**Figure 1B**); Magnetic resonance imaging (MRI)showed a cystic shape with long T1 and long T2 signals in the left posterior mediastinum. The shadow has a clear boundary and is approximately 1.8cm × 2.0cm × 3.8cm in size. It is considered to be a cystic lesion, possibly an enterogenic cyst. A mediastinal space-occupying resection was performed under thoracoscopic surgery. The operation went smoothly with 50 ml of bleeding and no blood transfusion was performed. After the operation, the patient returned to the ICU for monitoring and was given symptomatic treatment after the operation. He recovered well and was discharged from the hospital on June 19. Immunohistochemical staining(**Figures 1C–G**): The lining epithelium diffusely expresses estrogen receptor (ER), paired box gene 8(PAX8),Wilms’ tumor protein 1(WT1) and protein kinase c (PKC), is negative for calretinin, and has low proliferation of ki-67. Pathologic examination of tissue confirmed typical MCS histological features (**Figure 1H**).

**Figure 2:**
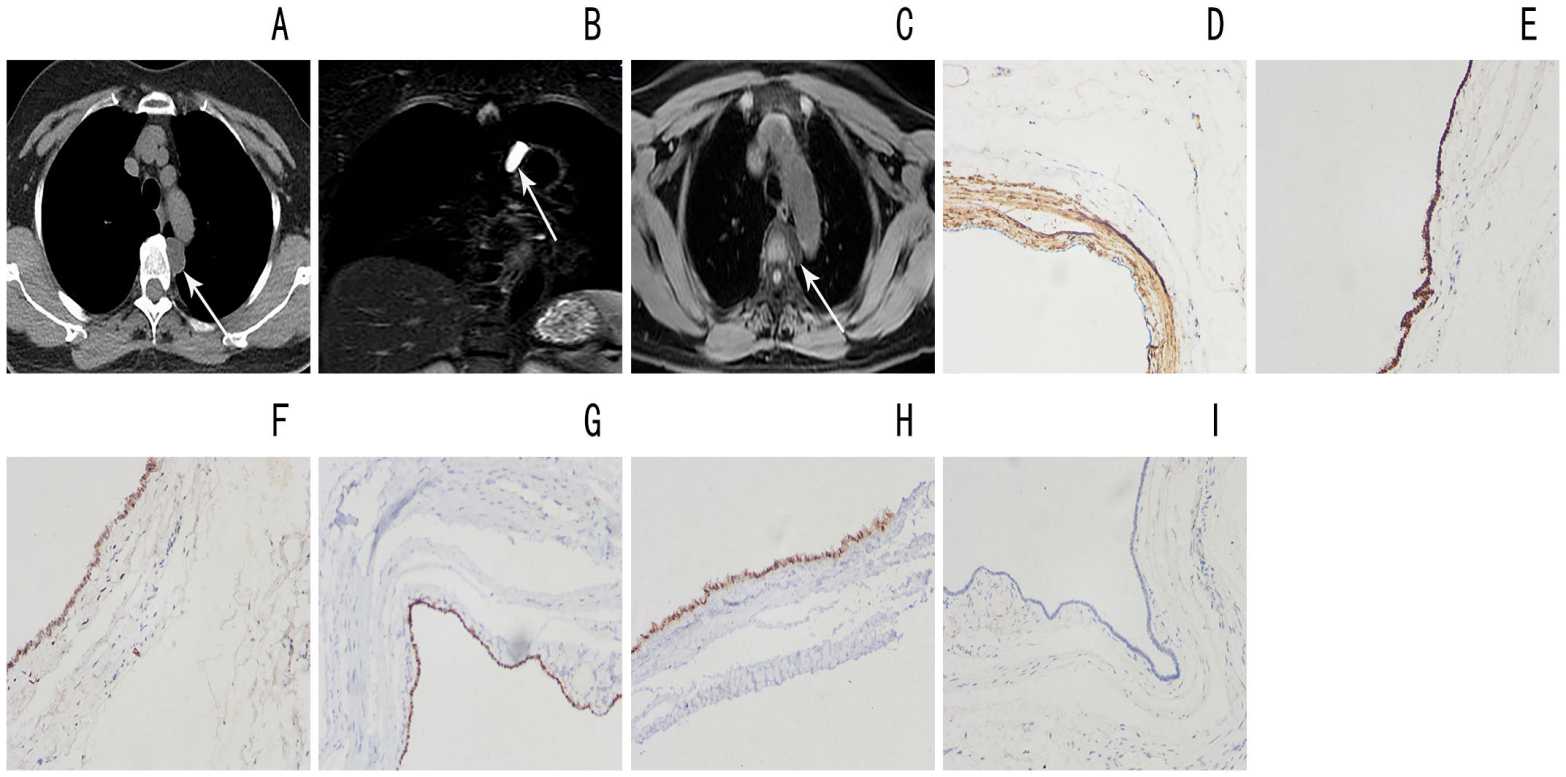
**(A)** Chest CT shows a low-density mass in the left posterior mediastinum with clear boundary (arrow) **(B, C)** Magnetic resonance imaging shows a long T1 long T2 cystic lesion in the left posterior mediastinum with well-defined borders (arrow) **(D)** Diffuse SAM positivity in the cyst wall lining epithelium High magnification **(E)** The epithelium lining the cyst wall is diffusely positive for estrogen receptor (ER) High magnification **(F)** The epithelium lining the cyst wall is diffusely positive for progesterone receptor (PR) High magnification **(G)** Diffuse WT1 positivity in the cyst wall lining epithelium High magnification **(H)** Diffuse PAX8 positivity in the epithelium lining the cyst wall High magnification **(I)** Low value-added Ki-67 in the cyst wall lining epithelium High magnification.

Case 2, female, 54 years old, went to the hospital on August 7, 2021 due to “pharyngeal discomfort when coughing up sputum”, with no other clinical symptoms; the patient’s blood pressure was high for 1 year, and was controlled by self-administered medication; the patient went to hospital about 3 and 6 years ago respectively. Thyroid surgery, physical examination showed old surgical scars on the neck; the patient’s body mass index was 22.3kg/m2. Chest CT showed a low-density round mass on the left front edge of the T4 vertebral body in the posterior mediastinum with a clear border and a CT value of approximately 18Hu (**Figure 2A**). Magnetic resonance imaging (MRI) showed that the lesion showed obvious long T1 and long T2 signals with clear boundaries and a size of approximately 1.5cm × 1.5cm × 2.5cm. It was considered to be a cystic lesion, an esophageal cyst or a Mullerian cyst (**Figures 2B, C**). The patient underwent mediastinal tumor resection under thoracoscopic surgery. The operation went smoothly, with 50 ml of bleeding and no blood transfusion. After the operation, the patient became critically ill and was transferred to the SICU. He was given ECG monitoring, oxygen inhalation and other treatments, as well as anti-infection, stomach protection, and detoxification. Symptomatic and supportive treatment such as sputum and nutritional support. Three days after the operation, the chest CT was reviewed and new infectious lesions in the chest were found, and symptomatic treatment was given. The chest tube did not drain much for three consecutive days, so the chest tube was removed. The patient’s condition gradually improved and he was discharged from the hospital on October 16. Immunohistochemical staining: The epithelium lining the cyst wall diffusely and strongly expressed SAM, progesterone receptor (PR), ER, WT1 and PAX8 (**Figures 2D–H**), was negative for calretinin, and had low proliferation of ki-67 (**Figure 2I**). The two patients did not receive any other treatment after surgery and recovered well. There was no recurrence after one year of follow-up.

## Discussion

Mullerian cysts (MCs) are related to residues during the development or degeneration of Mullerian ducts and are commonly found around the genitourinary organs or in the pelvis. However, there are only sporadic reports of MCs occurring in the mediastinum. This case has complete clinical/CT/MR/pathological data, which has good clinical guidance significance.

The pathogenesis of mediastinal MCs is unclear, and several theories have been proposed to explain the histogenesis of MCs in the mediastinum. Hattori (1) believed that cysts may originate from misplaced mesothelium and stroma and have characteristics similar to retroperitoneal MCs. Batt et al. (2) believed that the cyst was a choroidoma from the primary Mullerian organ, similar to the hypothesized pathogenesis of Mayer-Rokitansky-Kuster-Hauser syndrome reported by Ludwig (3).

Mediastinal MCs occur in the Th1-10 vertebral range of the por mediastinum and are concentrated in the T3-6 vertebral area (4). The age of onset is mainly in women aged 40-60 years (1, 5) osteri. Most mediastinal MCs develop during the perimenopausal period and are reportedly associated with obesity and various gynecologic histories, such as hormonal therapy, hysterectomy,artificial abortion and oophorectomy (4, 6). Therefore, hormonal abnormalities may be suspected to be involved in the development of mediastinal MCs. The 2 patients we report occurred at common ages (47 and 54 years old) and in classic locations (T4-5 vertebral level), on the left paraspinal side, and 1 patient had a gynecological history (history of breast fibroid resection), which is consistent with the literature. consistent with the description.

In clinical and imaging examinations, this type of cyst is usually mistaken for the more common bronchial cyst. Because it is located in the posterior mediastinum, it is also easy to mistake it for an esophageal cyst or schwannoma cyst; imaging (4, 5) Mediastinal bronchial cyst (Bronchial cyst, BCs) are usually composed of protein liquid. They appear as round or lobulated shadows on CT, with smooth edges, uniform cyst wall thickness, slightly higher density, and may have water-like density (CT value 0-20Hu) to high density (CT value 80-90Hu), slightly higher signal than the spinal cord on T1WI, while MCs usually contain serous and clear liquid, CT shows uniform low density, and T1WI signal is lower than the spinal cord signal; BCs due to increased intracystic pressure Tall and tend to be tense, showing a spherical structure, while MCs tend to show a flat structure (5, 7), this difference in shape helps to distinguish the two types of cysts.Histologically (5, 8), BCs are arranged with cilia, pseudostratification, columnar epithelium and goblet cells; the wall of BCs usually contains mucous gland tissue, smooth muscle and cartilage, and immunohistochemical staining of PAX-8, WT-1 and ER was negative. MCs are arranged in simple cylinders or cubes, are non-mucinous, and usually have ciliated epithelium, similar to uterine fallopian tube epithelium; immunohistochemical staining is positive for PAX-8, WT-1 and ER. Esophageal cyst is a type of enterogenic cyst, which is often located next to or within the esophagus, close to the location of the disease. However, it is more common in children and young people around 20 years old, and males are slightly more likely to be identified, and they can be combined. Congenital diseases such as esophagotracheal fistula and spinal deformity, and may contain protein to make the density slightly higher. The epithelium of enterogenous cyst can be lined by ciliated columnar epithelium as well, but can be differentiated by the occurrence of gastrointestinal tissue in the wall of enterogenous cysts. They rarely stain positive for ER, PR,WT-1 and PAX-8. The residual solid part of neurogenic tumors in the posterior mediastinum shows enhancement, while MCs appear as uniform thin-walled cysts with no enhancement or mild thin-walled annular enhancement (5), which is helpful to differentiate between the two.

Mediastinal MCs reported so far all follow a benign course, and recurrence has not been reported, but the long-term prognosis of MCs is still unclear. There is evidence that the presence of embryonic tissue, far from its typical location, may lead to malignant transformation (9), so surgical resection is the treatment of choice. Immunohistochemistry examination after surgery, such as PAX8, WT1, ER and PR positivity, can confirm the diagnosis. Due to the low incidence of mediastinal MCs, preoperative diagnosis is particularly difficult, and they are often diagnosed and treated clinically as bronchial cysts or other tumors. Therefore, in order to avoid misdiagnosis and reduce the risk of malignant transformation of Mullerian tissue, if cystic lesions with uniform watery density at the anterior edge of T3-6 vertebral bodies in the posterior mediastinum are found on imaging in middle-aged and elderly women, the possibility of this disease needs to be considered.

## Data Availability

The original contributions presented in the study are included in the article/supplementary material. Further inquiries can be directed to the corresponding authors.
